# Supplementary figures and images for: Improvement of Antialveolar echinococcosis efficacy of novel Albendazole-Bile acids Derivatives with Enhanced Oral Bioavailability
Source: PLoS Negl Trop Dis. 2023 Jan 3;17(1):e0011031. doi: 10.1371/journal.pntd.0011031 (PMC9838834; doi:10.1371/journal.pntd.0011031)

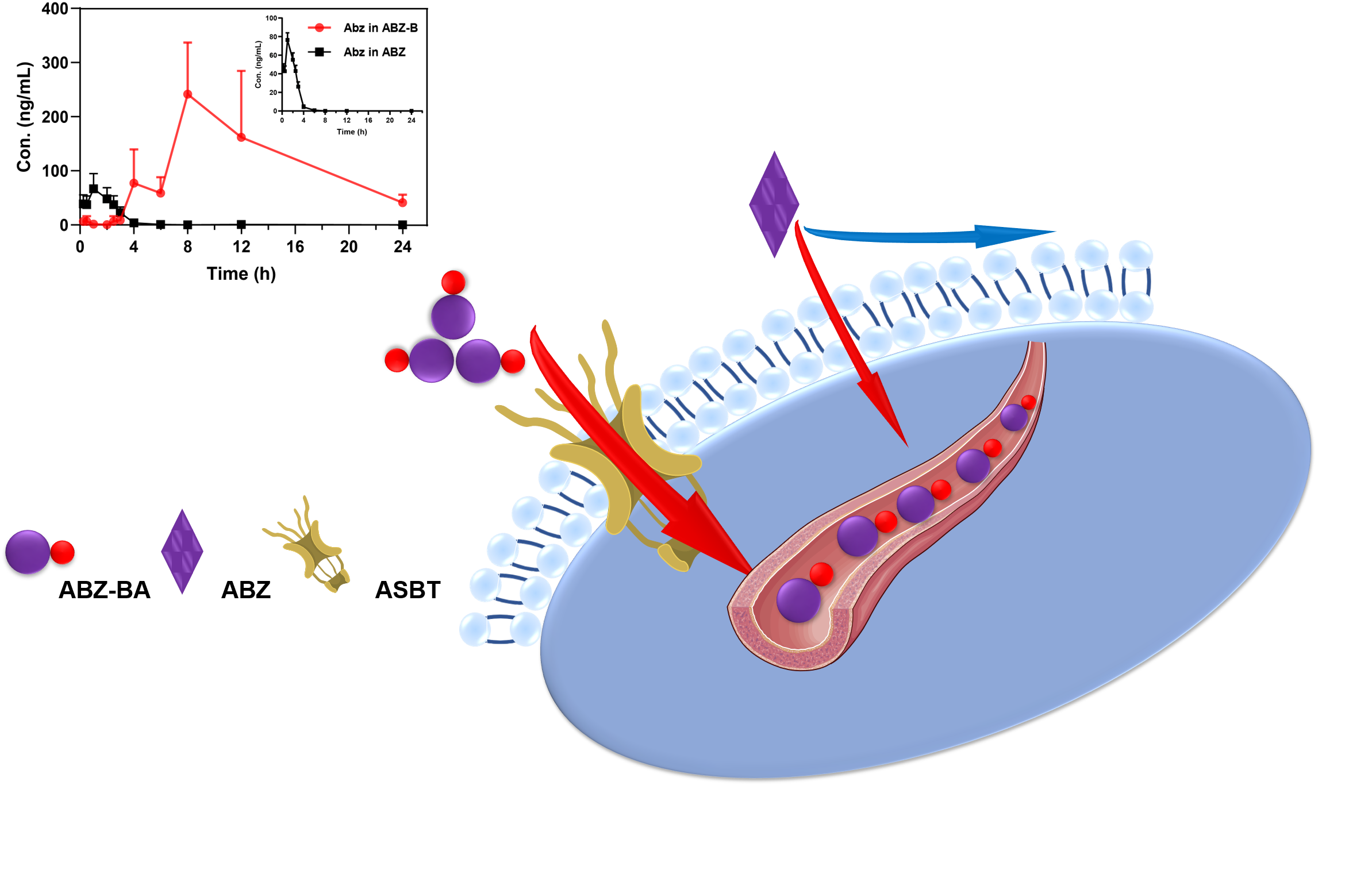

Supplement: S1 Fig — An optimized formulation containing Albendazole-bile derivative (ABZ-BA) was developed, which significantly improved the pharmacokinetics and the anti-AE efficacy, after a 30-day, once-daily oral administration. (TIF) [file pntd.0011031.s005.tif]

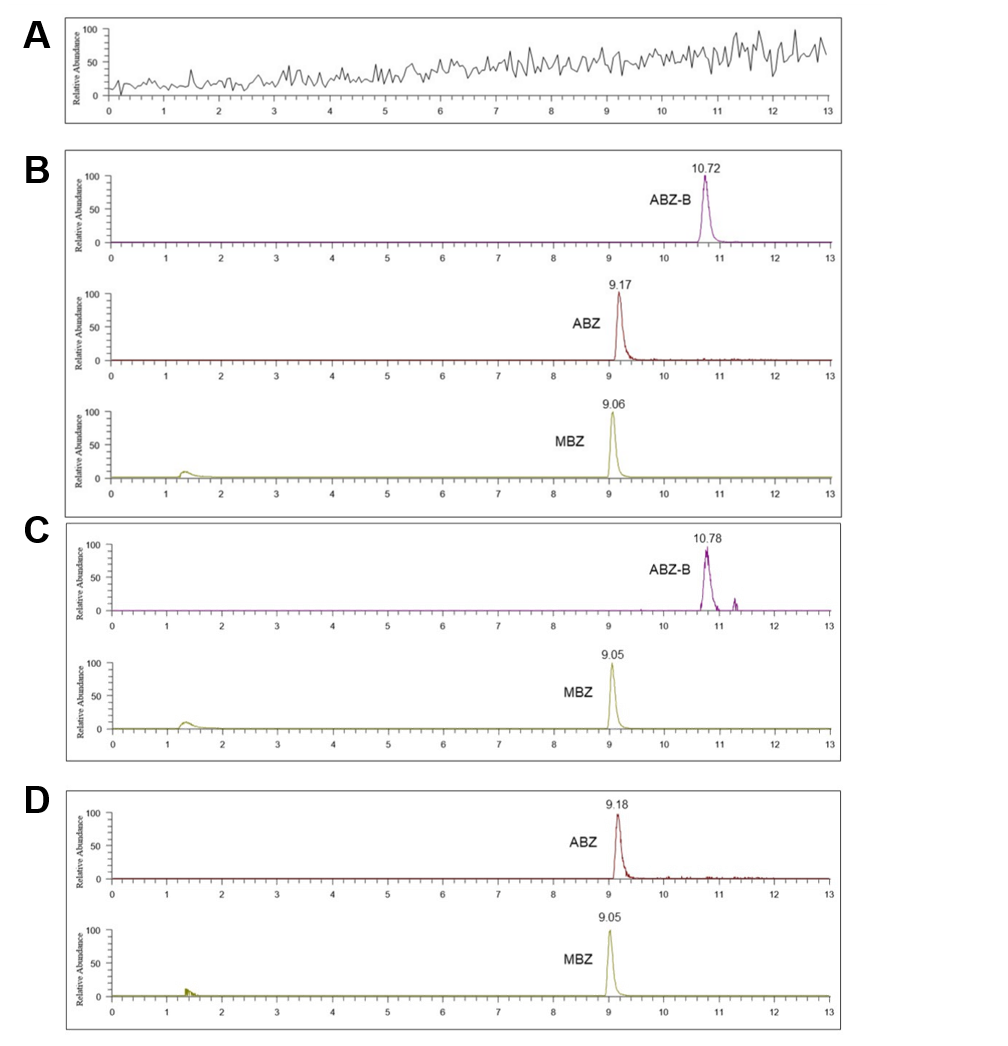

Supplement: S2 Fig — (A) Blank plasma; (B) Blank plasma +ABZ-BA and ABZ; (C) ABZ-BA and MBZ in plasma after oral administration; (D) ABZ and MBZ in plasma after oral administration. (TIF) [file pntd.0011031.s006.tif]
