# Supplementary material for: Improvement of Antialveolar echinococcosis efficacy of novel Albendazole-Bile acids Derivatives with Enhanced Oral Bioavailability
Source: PLoS Negl Trop Dis. 2023 Jan 3;17(1):e0011031. doi: 10.1371/journal.pntd.0011031 (PMC9838834; doi:10.1371/journal.pntd.0011031)
Supplement: Attachment — Submitted filename: Response to the Reviewers-20200926.docx [file pntd.0011031.s007.docx]

Response to the [Editor](javascript:;) Comments

Manuscript ID: PNTD-D-22-00677 (Improvement of Antialveolar Echinococcosis Efficacy of Novel Albendazole-Bile acids Derivatives with Enhanced Oral Bioavailability), Chunhui Hu, Meng Qin, Fabing Zhang, Ruixue Gao, Xuehui Gan, Tao Du

**Methods**
-Are the objectives of the study clearly articulated with a clear testable hypothesis stated?
-Is the study design appropriate to address the stated objectives?
-Is the population clearly described and appropriate for the hypothesis being tested?
-Is the sample size sufficient to ensure adequate power to address the hypothesis being tested?
-Were correct statistical analysis used to support conclusions?
-Are there concerns about ethical or regulatory requirements being met?
Reviewer #1: - Objectives are clearly stated
- The study design is appropriate
- The population is appropriate
- The sample size is adequate to address the hypothesis
- Correct statistical analysis is used
- There are no ethical concerns
Reviewer #2: (No Response)

Response: Thank you for the comment on my experimental methods of my manuscript.

**Results**
-Does the analysis presented match the analysis plan?
-Are the results clearly and completely presented?
-Are the figures (Tables, Images) of sufficient quality for clarity?
Reviewer #1: - The analysis relates with the study plan
- Results are clearly stated
- The tables and figures are clear
Reviewer #2: (No Response)

Response: Thank you for the comment on the experimental results of my manuscript.

**Conclusions**
-Are the conclusions supported by the data presented?
-Are the limitations of analysis clearly described?
-Do Wes discuss how these data can be helpful to advance our understanding of the topic under study?
-Is public health relevance addressed?
Reviewer #1: - Yes
- No
- This section needs elaboration
- Yes
Reviewer #2: (No Response)

Response: Thank you for the comment.

In the discussion, we have supplemented the limitations of the analysis of the modified method and explained how the resulting data can help promote readers' understanding of the research topic (L450-461).

**Editorial and Data Presentation Modifications?**
Use this section for editorial suggestions as well as relatively minor modifications of existing data that would enhance clarity. If the only modifications needed are minor and/or editorial, you may wish to recommend “Minor Revision” or “Accept”.

Reviewer #1: The language has some issues. Appropriate sentence structure is required at many places in the manuscript. Wes should also mention references where required.
Limitations of the study should be stated and there is no comparison of efficacy of this approach with other approaches used for this purpose. Even though, this approach presents a possible route to control the AE, there should be emphasis on its significance and its employability in comparison to the other studies.

Reviewer #2: (No Response)

 Response: Thank you for the comment.

We have used an English language editing service to revise the manuscript. Please ensure that any further comments on the language quality are as specific as possible.

We apologize for not giving a detailed description of its significance and employability in the manuscript. Since it improves a drug currently on the market, we emphasize the advantages of ABZ-BA in improving solubility and bioavailability compared with ABZ. However, the research is still in its early stages, so many questions have not been answered, including the role of ASBT in the transport of ABZ-BA and the feasibility of industrially producing ABZ-BA.

To further explain the advantages and limitations of the study, we have provided a detailed explanation in the discussion section of the new manuscript (L450-461)).

**Summary and General Comments**
Use this section to provide overall comments, discuss strengths/weaknesses of the study, novelty, significance, general execution and scholarship. You may also include additional comments for We, including concerns about dual publication, research ethics, or publication ethics. If requesting major revision, please articulate the new experiments that are needed.
Reviewer #1: The study is of significance as it represents one of the novel approaches for the treatment of AE which is affecting a huge chunk of human population. Data are well presented.
Reviewer #2: Alveolar echinococcosis (AE) is an orphan disease as well as a really neglected disease. Anti-AE therapeutic drugs are generally not a focus for pharmaceutical companies due to the economics or the lack of a viable market, and the drug R&D primarily depends on scientific researchers and NPOs. Although several efforts have been made in the last two decades, the anti-AE drug R&D is proceeding slowly; in contrast, the disease burden is still high. So far, albendazole (ABZ) remains the only drug treatment option, but suffers from the drawback of low oral bioavailability. In this context, discovering new chemical entities for the treatment of Echinococcus multilocularis infections should be encouraged. In the present work, Wes designed an albendazole bile acid derivative (ABZ-BA) with the purpose to improve the bioavailability of the parent drug ABZ through changing the crystal form and employing the sodium dependent bile acid transporter expressed in the small intestine. The study is designed reasonably. The chemical synthesis and structural characterization have been done thoroughly, but the work regarding to anti-parasitic activity as well as PK seems insufficient. The manuscript cannot be accepted unless the following major concerns have been addressed.

Major
1. The major drawback of the study is the lack of a therapeutic efficacy study in experimentally infected mice. According to the “Protoscolex collection and cultivation” section, Wes obviously have the access to E. multilocularis infection animal model. In vivo efficacy study must be accomplished before acceptance. It should be verified that whether the increased bioavailability of ABZ-BA claimed by Wes could translate to improved anti-AE treatment outcome.

Response: Thank you for the comment.

We also realized that the pharmacodynamic study of ABZ-BA against hepatic alveolar echinococcosis *in vivo* was the focus of this study, so we began to establish the *in situ* models of secondary hepatic alveolar echinococcosis in rats early on. According to the suggestions of the reviewer, we supplemented the cysts inhibition effect to demonstrate that ABZ-BA has superior *in vivo* pharmacodynamics compared with ABZ (L417-L458).

2. The in vitro anti-parasitic activity of ABZ-BA was only evaluated against protoscoleces but not metacestodes, while the latter stage is the primary parasite lesions in the intermediate hosts (e.g., humans and mice). The anti-parasitic ability of ABZ-BA against metacestode vesicles should be determined and compared with the parent drug ABZ to make sure that the structural derivatization did not exert negative impact on the activity.

Response: Thank you for the comment.

We hava added the evaluation of the microstructure of cysts to the in vivo pharmacodynamics experiment, including the evaluation of the effect of ABZ-BA on the germinal layer of cysts by scanning electron microscopy and HE staining. We believe that this data is more convincing than the *in vitro* against metacestodes study, and can better explain that ABZ-BA has better anti-Echinococcus properties than ABZ (L417-L458)).

3. Wes claimed that “sodium dependent bile acid transporter (ASBT) contributes for the enhanced bioavailability of ABZ-BA through effective transport of bile acids”, but no experimental evidence was provided.

Response: Thank you for the comment.

In the preface of the manuscript, we introduced two scientific hypotheses for designing ABZ-BA: one is based on the specific transport mechanism of bile acid by ASBT, and the second posits that bile acid can inhibit ABZ crystallization through steric hindrance.

In this manuscript, we only proved that the introduction of bile acid can effectively reduce the crystallization trend of ABZ. Additionally, we proved that ABZ-BA can effectively increase the dissolution rate and bioavailability of drugs compared with ABZ, and improved the anti-echinococcosis effect in vivo. For the specific transport of bile acid by ASBT, a significant amount of experimental data from the cell level to the animal level is needed to prove that the transmembrane transport of ABZ-BA is indeed related to ASBT. Our research group is performing research in this area and will publish the results soon.

Therefore, our goal in this manuscript is to prove the solubilization effect of ABZ-BA in vitro and in vivo from a macro perspective and to demonstrate the effect of bile acid on the crystallization trend of ABZ, without involving the transmembrane transport mechanism of ASBT on ABZ-BA.

Minor
1. Wes should add a statement why chose 4-amino-1-butanol as a linker? Is there a small-scale screening to identify an appropriate linker? If yes, please provide the screening design and results.

Response: Thank you for the comment.

We tested three linkers, including 2-amino-1-ethanol，4-amino-1-butanol, and 6-amino-1-hexanol. In the first reaction between Albendazole and linkers, the thin layer chromatography results showed that only 4-amino-1-butanol can form an obvious point, indicating a new product (intermediate 1). The MS and NMR results prove the correct structure of intermediate 1. Thus, we selected 4-amino-1-butanol as the linker. Since these experiments are all pre-experiments, there is no complete data and it cannot be provided in this manuscript.

 2. How many replicates were carried out to evaluate the protoscolecidal activity of ABZ-BA? In the terms of in vitro bioassay, at least three replicates are required.

Response: Thank you for the comment.

In fact, in the legend of Fig. 5, we have explained that there were three replicates in this experiment, but we forgot to add the error bars in Fig. 5A. We have added the error bars in the new manuscript.

3. For PK study, Wes should take into consideration the gender difference, please explain why only male rats were employed in the PK study. Also specify the formulations of ABZ and ABZ-BA for oral administration; I suppose both drugs are not water soluble.

Response: Thank you for the comment.

First of all, we only selected male SD rats in the PK study, in order to avoid the problem of data deviation caused by hormone changes in female rats. Moreover, in most published reports, male rats were also used for pharmacokinetic studies. In future experiments, we will pay attention to the influence of gender on our data, and use a 1:1 ratio of male to female rats to ensure the accuracy of the data.

ABZ and ABZ-BA are both water-insoluble, so we suspended the drug in a 0.5% sodium carboxymethyl cellulose (CMC-Na) aqueous solution. This method of administration has been widely used for the oral administration of poorly soluble drugs. This method has been adopted by our research group in previous studies, and many studies have been published that use this method, including the following:

(1) Improvement of the Bioavailability and Anti-Hepatic Alveolar Echinococcosis by Albendazole-Isethionate/Hypromellose Acetate Succinate (HPMC-AS) Complex. Antimicrobial Agents and Chemotherapy.

(2) Evaluation of Drug Dissolution Rate in Co-amorphous and Co-crystal Binary Drug Delivery Systems by Thermodynamic and Kinetic Methods. AAPS pharmscitech.

(3) Improvement of Antialveolar Echinococcosis Efficacy of Albendazole by a Novel Nanocrystalline Formulation with Enhanced Oral Bioavailability. ACS infectious diseases.

4. Have Wes evaluated the toxicity of the prepared ABZ-BA? Sometimes derivatization could result in elevated risk of toxicity. If not, I suggest cytotoxicity against human liver cells should be examined as least, as AE primarily affects the liver.

Response: Thank you for the comment.

We have used Hep-G2 cell line to evaluate the cytotoxicity of ABZ-BA and ABZ, and the results show that ABZ-BA with different carbon chain lengths (C1 refers to linker as 1 C; C4 refers to linker as 4 C) has the same cytotoxicity as ABZ, which proves that derivatization does not increase cytotoxicity. Since this part of data will be published in other manuscripts, it is not shown in this manuscript.

Fig.1 Cell viability of ABZ and ABZ-BA

5. The pharmacokinetic parameters, T1/2, clearance rate, F%, should be added into Table 5.

Response: Thank you for the comment.

We have supplemented relevant pharmacokinetic parameters in Table 1 of the new manuscript, including T_1/2_, clearance rate, and F%.

6. For Figures 3 and 4, make a clear statement that which kind of statistical analysis was used and add it into the figure legends.

Response: Thank you for the comment.

We have added specific statistical methods to the legends in Fig. 4 and Fig. 5 in the revised manuscript.

7. For Figures 6, Wes should not include the data of ABZ-BA, because only the plasma concentration of the parent drug ABZ matters for the comparison. The current figure design confuses the readers. Same problem in Figure 3A.

Response: Thank you for the comment.

We have deleted the data of ABZ-BA of Fig. 4 A and Fig. 6 according to your comments.

8. Figure 7 is needless, should be incorporated into Figure 1.

Response: Thank you for the comment.

We have merged Figure 1 and Figure 7 into a new figure, Figure 2.

9. Wes should provide the product information (cat#, manufacturer, place of production) for all reagents and materials. Some of the information is missing in the Methods section, please check it carefully.

Response: Thank you for the comment.

We have revised the product information for all agents and materials (L136-145).

10. Wes should provide the detailed method of isolation of E. multilocularis protoscoleces, since the protocol is complex, and cannot be simplified as the description “Protoscolex (PSC) of E. multilocularis was (not were) collected aseptically from alveolar echinococcosis obtained from the abdominal cavity of gerbils”.

Response: Thank you for the comment.

We have added detailed methods relating to the isolation of *E. multilocularis* protoscoleces in the methods section (L202-209).

11. Wes could put “Method validation of Ultra High-Performance Liquid Chromatography (UHPLC)-MS” section and its related methods into a supporting information file, which obviously is not the focus of the work.

Response: Thank you for the comment.

We have added a “Method validation of Ultra High-Performance Liquid Chromatography (UHPLC)-MS” section, along with its related methods, into a supporting information file.

12. A detailed ethic statement is necessary for the study, and makes it an independent section.

Response: Thank you for the comment.

As ethics statement has been added to the methods section (131-135).

13. The written English of the manuscript must be subjected to a professional language service and requires the editor’s extra attention.

Response: Thank you for the comment.

We have used an English language editing service to revise the manuscript. Please ensure that any further comments on the language quality are as specific as possible.
