# Supplementary material for: Improvement of Antialveolar echinococcosis efficacy of novel Albendazole-Bile acids Derivatives with Enhanced Oral Bioavailability
Source: PLoS Negl Trop Dis. 2023 Jan 3;17(1):e0011031. doi: 10.1371/journal.pntd.0011031 (PMC9838834; doi:10.1371/journal.pntd.0011031)
Supplement: Attachment — Submitted filename: Response to the Reviewers-20221117.docx [file pntd.0011031.s008.docx]

Reviewer #1: The manuscript has greatly improved in terms of clarity, understanding and English language.
Just a few suggestions are given for correction of the sentences:
L70: Omit ‘of the’

**Response:** Thanks for pointing this out. We have omitted ‘of the’ in L70.
L 75: Echinococcus multilocularis should be in italics

**Response:** Thanks for pointing this out. We have modified ‘Echinococcus multilocularis’ to italicize in L75.
L 85: multilocularis is the name and it should be written in its full form

**Response:** Thanks for pointing this out. We have changed the word “multilocula” to “multilocularis” in P85.

L 86: humans and animals

**Response:** Thanks for pointing this out. We have changed the word “humans or animals” to “humans and animals” in P86.
L 94-96: reference/s missing

**Response:** Thanks for pointing this out. We have supplemented references.
L 97-98: This is repetition of what you have stated in opening paragraph

**Response:** Thanks for pointing this out. We have deleted this sentence.
L 120: Rephrase this sentence for more clarity

**Response:** Thanks for pointing this out. We have rephrased this sentence.
L 166: bile instead of bill

**Response:** Thanks for pointing this out. We have changed the word “bill” to “bile” in P166.
L 327: ABZ-BA
**Response:** Thanks for pointing this out. We have changed the word “ABZ-Ba” to “ABZ-BA” in P327.

Reviewer #2: I am glad to see the authors have made efforts to improve the scientific quality of the manuscript. Before acceptance, there are still a few concerns to be addressed.
1. One of the issues is the newly added “Pharmacodynamic evaluation” section. I suppose all the drug formulations were orally given, as it is the proper way to prove the hypothesis of bioavailability improvement, but the authors gave a statement of administration with “injection”. Please give an explanation.

**Response:** Thanks for pointing this out. I'm very sorry, this is our writing error. We use intragastric administration. Because we use a syringe for intragastric administration, we mistook oral administration as injection.

--How long to initiate the drug administration after infection?

**Response:** Thanks for pointing this out. The drug was administered at the 16^th^ week after infection, and we have supplemented relevant instructions in L290.
--There is no clear statement of animal housing conditions, animal grouping, and administration period in the method section.

**Response:** Thanks for pointing this out. We have supplemented the animal housing conditions (L287-289), animal grouping (L293-296) and administration period (L299) in the manuscript.
--The description of “pharmacodynamic evaluation” is not a common use, “in vivo therapeutic efficacy in an in-situ AE animal model” would be better.

**Response:** Thanks for pointing this out. We have changed the word “pharmacodynamic evaluation” to “in vivo therapeutic efficacy in an in-situ AE animal model”.
2. The other concern is the statistical analyses. Comparisons between two groups should use the t-test. For comparisons among multiple groups (≥ 3), one-way ANOVA followed by a reasonable post-hoc test (e.g. Tukey’s) or a nonparametric test (e.g. Kruskal–Wallis) should be used. For example, t-test for the comparison of two groups ABZ and Abz in Fig. 4A, and one-way ANOVA or Kruskal–Wallis test for the three groups in Fig. 7D. The authors should recheck the statistical analysis throughout the manuscript.

**Response:** Thanks for pointing this out. We rechecked the statistical analysis of the entire manuscript and corrected the errors found.
3. There is no oral bioavailability F% in Table 1, which is a crucial parameter.

**Response:** Thanks for pointing this out. We have supplemented the relative bioavailability (F_rel_) in Table 1.
4. The improvement of F% and the reduction of cyst volume should be highlighted in the abstract and author summary.

**Response:** Thanks for pointing this out. We have supplemented the F% and the reduction of cyst volume in the abstract and summary
5. For Fig. 5A, 5D and 5E, replace the legends of Group 1-7 with drug names and concentrations.

**Response:** Thanks for pointing this out. Since the composition of the culture medium is relatively complex, marking it directly on the diagram will destroy the overall format and aesthetics of the diagram, so we added Table 1 to explain the drug, concentration and composition.
6. Change all the mass concentrations to molar concentrations in Figures 4-6 and Table 1, easy to make a comparison of ABZ and ABZ-BA.

**Response:** Thanks for pointing this out. Since various ABZ derivatives, such as ABZ-BA and ABZ-SO, are used in this study, we will directly convert the mass of the substance through the mass of ABZ. However, if all the ABZ derivatives are converted to molar mass, readers will have a little difficulty in reading. In addition, according to the practice of publishing articles, we rarely see articles that describe the drug concentration time curve and pharmacokinetic parameters with molar mass, so we do not recommend changing the drug mass to molar mass. However, if PLoS Neglected Tropical Diseases has special requirements, we are willing to accept the suggestions of experts and editors.
7. The language editing requires extra attention of the editors.

**Response:** Thanks for pointing this out. We have corrected the language editing in the manuscript according to the suggestions of the reviewer.
